# Type of opioid dependence among patients seeking opioid substitution treatment: are there differences in background and severity of problems?

**DOI:** 10.1186/s13011-016-0066-1

**Published:** 2016-07-11

**Authors:** Bodil Monwell, Per Bülow, Arne Gerdner

**Affiliations:** Department of Dependency, Psychiatric Clinic, County Hospital Ryhov, Jönköping, Sweden; Jönköping University, School of Health Sciences, Jönköping, Sweden; Psychiatric Clinic, County Hospital Ryhov, SE-551 85 Jönköping, Sweden

**Keywords:** Opiates, Opioids, Opioid-related disorders, Opioid Substitution Treatment

## Abstract

**Background:**

The study explores differences and similarities in background and problem severity among those seeking Opioid Substitution Treatment (OST), comparing those who primarily had misused "opiates", e.g. heroin, morphine and opium, with those who primarily had misused other opioids.

**Methods:**

Patients (*n* = 127) assessed for possible admittance in OST are compared based on the Addiction Severity Index. Two groups based on primary type of opioid misused are compared (opiates vs. other opioids).

**Results:**

In the global severity ratings there were no significant differences between the groups other than tautological artefacts concerning heroin. There were few specific differences between the groups. The opiate group more often had Hepatitis C and more often had legal problems related to financing their misuse. Injection of drugs was the main method of administration in both groups, i.e. 90 % for mostly opiates vs. 75 % for mostly other opioids. A great majority in both groups, 96 % vs. 91 %, had misused most other types of drugs. Both groups were found to have severe problems in all areas investigated.

**Conclusions:**

The study demonstrates great similarities in problem severity among those seeking OST, both those who primarily had misused opiates and those who primarily had misused other opioids.

## Background

Opioid dependence is a serious brain-related chronic disease with a high risk of premature death [1–4]. Opioids can be used as painkillers and for other purposes based on assessment by, and prescription of, a physician, or they can be misused for getting high and other reasons without prescription and against medical advice. Here the term ‘opioid misuse’ always refers to use without prescription or against medical advice. Opioid misuse occurs worldwide. In 2012, the estimated prevalence in the age group 15–64 years was between 26 and 36 million, of which 50 % were misusing “opiates”, particularly heroin [5]. There are currently a large number of opioid drugs that are misused and can lead to dependence, and the number of new synthetic drugs is rapidly increasing [5, 6]. In 2012, it was reported that heroin was partially being replaced by fentanyl and other opioids in the European drug market [6]. In North America and Oceania there is an increasing problem with people becoming dependent on other kinds of opioids than heroin [5, 7–11].

There is some confusion concerning the concepts of opioids and opiates. ‘Opiates’ refer to the natural derivatives of opium, while the main term ‘opioids’ includes both opiates and all other opioids with similar effects [12–14]. All opioid substances activate the opioid receptors in the brain [15, 16]. Since the 1970s, drug dependencies have been considered as diseases according to the diagnostic systems ICD (International Classification of Diseases) and DSM (Diagnostic and Statistical Manual of Mental Disorders) [12, 13]. It should be clarified that the diagnostic term for dependence on opioids in both international diagnostic systems is opioid dependence (F11.2 in the ICD-10 and 304.00 in the DSM-IV), regardless of whether the substances misused are heroin, morphine, opium or any other kind of opioid (e.g. fentanyl, ketobemidone). As the negative effects of substance use and dependence are considerable, effective treatment is a matter of great concern. Opioid Substitution Treatment (OST) is a pharmacological treatment with methadone or buprenorphine and is preferably administered in combination with psychosocial treatment and support interventions [17–21]. OST has been extensively researched and has been shown to benefit both individuals and society by reducing drug misuse and the risk of premature death, and by promoting retention in treatment, and social rehabilitation [3, 18, 19, 21–24]. OST was, in 2010, available in 77 countries [23]. The WHO guidelines (2009) clearly addresses the treatment to opioid dependency, based on the diagnosis according to ICD-10 or DSM-IV, i.e. it does not depend on which type of opioid substance misused [21].

Sweden is an exception to this. Sweden is traditionally a very restrictive country when it comes to drug policy. Although methadone maintenance treatment was introduced in Sweden already in the 1966, as the first country outside US, it was always regarded as an exception since it did not comply with the political slogan “a drug-free” society. The restrictive policies have gradually shifted and OST is now regarded as a recommended treatment. However concerns are still strong against “leakage” i.e. prescribed drugs from the program sold at the illegal market, and against having broad eligibility to the programs, and these concerns contributed to the formulation of the Code of Statutes in a problematic way. In March 2010, the Code of Statutes (SOSFS 2009:27(M)) was altered and patients dependent on other opioids were no longer eligible for admittance to OST. Only those dependent on “opiates” – defined in the Code as three of the opium alkaloids: heroin, opium and morphine – were admitted from that date. The decision was not accompanied by any studies indicating differences in needs for treatment and care between the groups [25]. It was mentioned that there was a lack of evidence concerning the effectiveness of OST for other than those “dependent on opiates”. However, the SBU (The Swedish Council on Health Technology Assessment) concluded from a systematic review [26] in 2009 that OST is an effective treatment regarding opioids, not distinguishing heroin, opium and morphine from other opioids.

In Sweden 2010, the average age of death, with presence of heroin, morphine or methadone found at autopsy, was 36 years [27]. This problem was discussed by the Government Task Force on Substance Misuse, which in 2011 reported that about 10,500 persons in Sweden were “addicted to opiates”. However, the term “opiates”, that was used in a cited report, included all kinds of opioids as well as natural derivatives (an example of the confusion mentioned above). There is no epidemiological study that specifies what kinds of opioids are used in Sweden. The National Board of Health and Welfare (SoS) estimated in February 2014 that about 3,700 people received OST at about 90 OST units (personal communication with Ulf Malmström, SoS). Thus, the majority of those dependent on opioids are not admitted to OST. There is now a great variety of poly drug use, administration patterns and specific mixtures of preparations, increasingly characterized by a variety of opioids combined with other drugs [28]. Such patterns may complicate OST, and therefore have clinical importance [28–32]. “Pure” heroin addicts are less common, raising new demands for care and treatment [5, 7, 28]. There are several studies comparing different subgroups of misused opioids but none is comparable to the Swedish categorization of opioids [9, 30–33]. Previous research has mainly focused on heroin dependency. Although there are studies on opioid dependency among those using “prescription opioids” and “opioid-analgesics”, those terms have different meanings in different publications – and in different countries [32–34]. The aim of this study is to compare, based on type of opioid used, the background and problem severity of groups of persons who were assessed for OST.

The following three questions will be addressed: a) Are there differences in the severity of drug problems or in related problem areas (e.g. alcohol, health, social situation)? b) If so, are the differences of clinical significance? c) What is the situation specifically for those who were categorized as opiate users in relation to those who were categorized as users of other opioids?

## Methods

This was a naturalistic study conducted at a Swedish county hospital, and concerns persons assessed for OST admittance in 2005–2011. In 2005, a manual-based OST assessment was introduced at the clinic, supplemented with ASI (Addiction Severity Index) interviews carried out by referring municipal social boards. The OST assessment was based on a thorough investigation of documentation such as medical journals and police registers, as well as a semi-structured interview. The interview determined descriptive details of the social situation, background, and medical and psychiatric status (e.g. other illnesses) of the client. It also included a comprehensive history of what kind of substances the person had used and misused and which of these dominated. This included a breakdown of all types of opiates and other opioids, as well as other substances used, the duration and amount of doses of each drug, the mixture of drug preparations, administration patterns, experiences of overdoses and how these were handled etc. The first author (BM) conducted all assessments after 2005 following the same procedure.

The ASI is a valid and globally used structured interview that estimates problem severity and need of help in seven areas: medical status, employment and support status, drug use, alcohol use, legal status, family and social relationships and psychiatric status [35]. For these seven areas, three different types of measurements are used. First, there is the interviewer severity rating which can range from 0 (= no problems/no help needed), to 9 (= life threatening problems/in urgent need of help). The second is the self-assessment of needs, which can range from 0 (= no problem/no help needed) to 4 (= very great problems/assistance definitely needed). The third estimate is the number of days, within the last 30, on which these problems were experienced, varying from 0 to 30.

Before a patient is referred to OST, the ASI interview with the person in question is usually carried out by the referring municipal social worker. The social worker sends the clinic the social assessment concerning the person, including the ASI interview, along with a proposed treatment plan for psychosocial interventions to supplement the medical treatment. During the study we found that different versions of the ASI had been used. Careful examinations of the differences in wordings of specific items were made, and 11 items with significant changes in meaning were excluded from the analysis. Thus, the data from the ASI interviews used here are comparable, regardless of version.

The data on various problems presented in this article are based on the ASI interviews, while the categorization into the two groups, i.e. those categorized as having primarily misused opiates vs. those categorized as having primarily misused other opioids, is based on the OST assessment. Eligibility for OST treatment– according to Code of Statues for OST – must be validated by documentation from e.g. hospital and police records and/or drug tests, and thus should not be based only on interview material.

The total number of patients considered for OST during 2005-01-01 to 2011-06-30, was 153. Of these, 26 patients were transferred from another OST unit or from a pain unit. These patients were excluded from the present study since they were not assessed according to clinical routines. Of the remaining 127 persons, 80 individuals (63 %) were categorized as having opiates as their dominant drugs while 47 persons (37 %) were categorized as having other opioids as their dominant drugs of misuse. Although this paper concerns differences in problems and needs based on the ASI, it should be noted that those categorized as users of other opioids had all (100 %) lived in the Jönköping county or in rural areas in nearby counties for a very long time, while 45 % of those categorized as opiate users had either migrated from another country, or from larger cities in other parts of Sweden where heroin misuse had been established for a longer time.

For 17 of the 127, it was found that the complete ASI forms were not available and could not be obtained from the municipal social services – only compiled reports based on the original interviews were available. In order to get complete data sets, these 17 individuals were asked to participate in an ASI interview retrospectively, focusing on the situation at the time they were actualized for admittance to the OST program. Thus, the 127 ASI interviews include 110 prospective and 17 retrospective interviews (14 in the opiate group and three in the other opioids group). The retrospective interviews were then compared with the OST-investigation – including the compiled reports of the original ASI interviews – and with other hospital records, and found to be highly in agreement. As a safeguard however, comparisons between the opioid type groups were repeated without using the retrospective interviews. That did not change any findings though it weakened the statistical power due to there being fewer cases.

Differences in mean values were tested for statistical significance using a Student's T-test for two independent groups. Kruskal-Wallis H-test was used when comparisons were made with ordinal and skewed interval scales. When frequencies were compared, a Chi-2 test and Fisher's exact test were used. The five per cent significance level was applied.

Since the ASI data in this study enables a very large number of group comparisons–in this case 249 variables–there is an obvious risk of mass significance. That could result in a risk of getting 249 × 0.05 = 12 differences labelled as significant based solely on chance, i.e. without real differences existing. This problem justifies the use of a correction method. The Bonferroni correction method multiplies p-values by the total number of comparisons, here possibly 249 comparisons. In practice, it would be impossible to find significant values with such a method. The Bonferroni method therefore seems to be too restrictive and it might hide existing group differences. This study therefore used a pragmatic strategy involving two methods. First, the tables show crude p-values, i.e. without applying any correction method. Secondly, the Bonferroni-Holm correction method was applied. Thus, the problem of mass significance could be limited without hiding possible real differences. In addition to the use of p-values, 95 % confidence intervals of differences in mean values and boxplots of distributions of ordinal scaled indexes were used in order to assess the degree of overlapping distributions between the groups. Both these were explored to detect possible differences of clinical relevance for treatment and rehabilitation.

The study has been subjected to ethical review and was approved by the Regional Ethical Review Board in Linköping, 2011-06-15 (2011/214-31) and 2014-01-13 (2013/497-32).

## Results

Table 1 presents the background characteristics and demographic data of the two groups, i.e. those categorized as having opiates vs. those having other opioids, as their main types of drug.

**Table 1:** Comparison of background and demographics based on ASI inter-views for two groups: those who dominantly used opiates vs. other opioids

Total	Opiates	Other opioids	Crude *p*-values
*n* = 127	81	46	
Gender, woman, %	14	20	.37 (a)
Age, m (sd)	36.5 (10.3)	35.4 (9.9)	.49 (b)
In controlled environment during the last 30 days, %			.20(a)
- No	58	70	
- In drug-free residential or detox %	28	15	
- Other	14	15	
Number of days in controlled environment last 30 days, m (sd)	6.19 (10.5)	4.91 (10.3)	.52 (b)
Ethnicity, %			.53 (a)
- Swedish	88	91	
- Other	12	9	

M (sd) = Mean (standard deviation). Significance tested by: a) Chi-2, b) T-test for independent groups

The table indicates that no significant differences between groups were detected. Both groups had used the majority of opioids even if they had an “opioid of choice” which – from the necessity of the code of statutes – placed them in one of the groups.

### Severity of problems in seven areas

Table 2 presents the severity of the seven problem areas, based on the interviewers’ severity ratings, patients' self-ratings of problem severity, and the number of days within the last 30 that patients reported having had these problems.

**Table 2:** Comparison of global severity in seven problem areas, based on the interviewers’ and self-assessed ASI severity ratings, and number of days with the problem, for two groups of opioid addicts, based on main type of opioid misused

Severity of problems	*n* = 127	Opiates	Other opioids	Crude
	81/46^a^	81	46	*p*-values
Interviewers’ severity ratings, mean ranks:				(a)
Medical status	67/43	55.3	55.8	.946
Employment/support status	66/43	53.0	58.1	.395
Alcohol status	69/43	56.7	56.2	.941
Drug status	69/42	60.9	47.9	.034
Legal status	66/43	56.4	52.8	.527
Family/social relationships	67/42	53.3	57.8	.459
Psychiatric status	66/42	53.3	54.8	.944
Clients’ severity ratings, mean ranks:				(a)
Medical status	76/46	60.8	62.7	.762
Employment/support status	75/45	59.2	62.7	.559
Alcohol status	78/46	63.2	60.0	.495
Drug status	77/46	66.3	54.8	.034
Legal status	76/46	62.3	60.1	.704
Family/social relationships	78/46	60.8	65.4	.471
Psychiatric status	77/45	57.5	68.3	.096
Number of problem days in last 30, m (sd):				(b)
Medical status	77/46	14.1 (13.5)	15.3 (12.7)	.232
Employment/support status	77/42	8.2 (13.0)	6.0 (11.4)	.053
Alcohol status	79/45	3.4 (9.2)	2.2 (6.9)	.095
Drug status	79/45	25.4 (10.0)	20.7 (12.8)	.036
Recent illegal activities. for profit	77/44	5.0 (10.0)	1.3 (5.0)	.007**
Relationship problems	77/44	5.8 (10.9)	4.5 (9.0)	.056
Psychiatric status	73/44	15.9 (13.2)	16.6 (13.6)	.638

Significance tested by: a) Kruskal-Wallis H test, and b) T-test for two independent groups

^a^Opiates group/Other opioids group. **Significant after Bonferroni-Holm correction

The average rankings (mean ranks)

In five of the seven areas there were no significant differences in any of the three global measures. Differences were found in two areas: drug status and recent illegal activities. All three measures detected a more severe drug problem in the opiate group. However, those differences in drug status did not hold when the Bonferroni-Holm correction was applied. Another difference concerned the number of days spent on illegal activities for profit during the preceding 30 days. That finding was also robust when applying the correction method. These differences will be further explored in the next section. The boxplot, however, showed that the boxes as well as the arms totally overlapped between the comparison groups. Hence, no differences of clinical significance were indicated. Further comparisons on substance use problems are explored since differences between the two groups concerning drug problems were found in all three measures, the results of a further investigation of substance use problems on the level of specific items are given in Table 3. The investigation concerned what specific substances were used, indications of particularly hazardous use, e.g. injection and overdose, duration of misuse, and whether misuse had led to treatment.

**Table 3:** Comparison of persons who had used opioids assessed for OST, divided into two groups regarding substances used, for how long, risky behaviour, and previous treatments, based on ASI interviews

	n = 127	Opiates	Other opioids	Crude
	81/46^a^	81	46	*p*-values
Substances used, %				(a)
Alcohol over threshold	80/45	78.8	84.4	.438
Heroin	80/45	96.3	64.4	<.001**
Methadone	78/45	78.2	66.7	.160
Other opiates/analgesics	80/46	97.5	100	.280
Medicines/pills (sedatives)	80/46	91.3	91.3	.992
Cocaine	78/45	71.8	51.1	.021
Amphetamines	80/45	92.5	91.3	.811
Cannabis	80/45	92.5	77.8	.018
Hallucinogens	77/44	72.7	56.8	.074
Inhalants	77/44	42.9	31.8	.231
Other	76/42	57.9	57.1	.937
Hazardous use				
Multiple substances, %	75/43	94.7	90.7	.387
Injection, %	81/46	90.1	73.9	.016
Overdoses, m (sd)	77/44	5.0 (6.6)	3.1 (5.8)	.219 (b)
Duration of use, years, m (sd)				(b)
Alcohol over threshold	61/36	8.3 (9.6)	7.0 (8.3)	.158
Heroin	72/23	8.6 (7.8)	1.7 (3.1)	<.001**
Methadone	48/23	2.3 (5.0)	1.2 (2.5)	.245
Other opiates/analgesics	72/39	8.9 (8.1)	10.7 (9.5)	.164
Medicine/pills (sedatives)	68/37	9.0 (9.2)	9.0 (8.0)	.245
Amphetamines	66/35	9.0 (9.3)	4.8 (4.8)	.004**
Cannabis	66/33	11.5 (9.7)	6.3 (5.3)	.001**
Hallucinogens	45/20	0.6 (1.1)	1.0 (2.6)	.475
Inhalation	27/12	1.2 (2.2)	0.3 (0.6)	.093
Others	33/20	0.9 (1.7)	2.9 (6.3)	.188
Multiple substances	66/34	12.4 (8.9)	9.7 (8.5)	.598
Injection	67/29	11.4 (9.4)	7.8 (8.9)	.806
Treatments, m (sd)				
Alcohol problems	80/45	9.3 (46.8)	5.1 (12.8	.226
Alcohol detox	73/43	4.1 (17.1)	2.5 (6.3)	.159
Drug problems	80/40	14.7 (45.1)	9.7 (11.7)	.426
Drug detox	73/42	6.4 (13.2)	6.3.(7.8)	.785

Significance tested by a) Chi-2 if not otherwise stated, and b) Students T-test for independent groups

^a^Opiates group/Other opioids group. ** Significant after Bonferroni-Holm correction

Two of the significant differences concern heroin (percentage using, and duration). Although these differences were confirmed after Bonferroni-Holm correction, they are tautological artefacts. They may look like findings, but are just consequences of the categorization, since heroin is the main opiate. Two other findings concerned longer duration and earlier age of onset of cannabis and amphetamine misuse in the opiate group. In addition to these, there were some findings that did not hold for the correction, e.g. more persons injecting in the opiate group. However, it should be noticed that those who mainly misused other opioids also had remarkably high rates of injection (about 74 % compared to about 90 % for the opiate group). More than 90 % of both groups used multiple substances, and the two groups had comparable numbers of overdoses. In addition, both groups had multiple treatment episodes for various substances.

### Detailed comparisons concerning health and social situation

Table 2 above shows great similarities in severity in all problem areas except drugs. Still, there might be differences in specific problems that could be relevant when deciding on the need for treatment and support.

The last table therefore shows comparisons between the groups for health and social situation, in order to explore if there are variations that might justify differences in treatment policies.

As shown in Table 4, a great majority in both groups have chronic medical problems. There are no significant differences between them neither regarding chronic medical problems, nor in having the status of disability pensioner due to such problems. However, there is a difference in one specific medical problem, since those who misused opiates more often suffer from hepatitis C. As for psychiatric health, there are no differences between the groups in specific problems such as anxiety, depression and suicidal thoughts or acts. They are victims of emotional, physical and sexual abuse to the same extent. The groups are also similar in terms of social situation, with very few cohabitating or being wage-earning employees. In Table 2, we find a difference concerning the number of days during the last 30 spent on illegal activities for profit. The opiate group has a significantly higher rate of self-reported crime with the aim of making money. This was expected based on the much higher prices of heroin on the illicit drug market compared to other opioids, e.g. fentanyl. However, there are no significant differences between the groups regarding the number of times in life they report having been charged with drug-related crimes, crimes of property (i.e. robbery/theft), crimes of violence, driving drunk or doped, or other crimes, e.g. vandalism. Both groups had been detained for drug offenses. The only significant difference concerning crimes in Table 4 is that the opiate group score higher on having been apprehended for serious traffic crimes. There are also no differences between the groups concerning legal status, living with someone who has problems with alcohol or drugs, the people with whom they spend their leisure time, and how satisfied they are with the situation (those variables are not shown in the tables).

**Table 4:** Comparison of health and social situation from ASI for persons who misused opioids, divided into two groups, opiates vs. other opioids

	*n* = 127	Opiates	Other opioids	Crude
	81/46^a^	81	46	*p*-values
Medical status %:				(a)
Chronic medical problem	81/46	70.4	78.3	.334
Hepatitis C	81/44	66.7	38.6	.003**
Income from health insurance for medical problems	81/45	19.8	20.0	.973
Sources of financial support last 30 days %:				
Health insurance	80/46	38.8	50.0	.219
Employment	80/46	17.5	8.7	.174
Psychiatric problems, previous experienced %:				
Serious depression	79/46	73.4	84.8	.142
Serious anxiety	78/46	79.5	87.0	.293
Serious suicide thoughts	77/43	61.0	74.1	.138
Suicide attempts	76/44	2.0	1.6	.150
Victim of emotional abuse	77/42	64.3	66.2	.831
Victim of physical abuse	76/43	55.3	58.1	.761
Victim of sexual abuse	76/42	23.7	21.4	.780
Treatment for psychiatric problem in hospital, m (sd)	78/45	2.7 (6.2)	2.5 (6.3)	.691(b)
Legal problems, m (sd): Type of charges				(b)
- Drug-related crimes	77/38	6.7 (12.9)	3.4 (10.0)	.550
- Crimes against property	76/39	7.7 (17.1)	4.1 (9.3)	.136
- Crimes of violence	77/42	2.6 (7.1)	1.9 (3.7)	.331
Apprehended for				
- Driving drunk/doped	76/42	1.8 (3.9)	1.0 (1.9)	.145
- Serious traffic crimes	76/41	3.6 (7.7)	0.7 (1.9)	.002**
- Other crimes	77/39	2.4 (7.3)	1.4 (4.9)	.239
Social status %:				
Housing residence	81/46			.840 (a)
- Own housing contract		61	61	
- Other housing		33	30	
- Homeless		6	9	

M (sd) = Mean (standard deviation). Significance tested by: a) Chi-2, b) T-test for independent groups

^a^Opiates group/Other opioids group. ** Significant after Bonferroni-Holm correction

## Discussion

All individuals in the study applied for OST when they felt they had extensive problems with an opioid dependence. The main finding of this study is that there are great similarities between the groups concerning severity of drug related problems as assessed by ASI. This is consistent with the fact that both groups, in everyday clinical practice, were perceived to have extensive needs for care. The study does not examine severity of opioid dependence, in terms of diagnostic criteria (this will be focus in a coming article), nor does it explore all clinical differences between specific opioid substances. Instead, its focus is on the relevance of the categorization in two groups of opioids, i.e. opiates vs. other opioids as the bases for OST eligibility. In most problem areas, severity according to self- and interviewer ratings and number of days with a problem are similar when those who used opiates and other opioids, respectively, are compared. The main difference concerns drug problems, but a closer look at that problem area shows that most differences are in fact tautological artefacts concerning misuse of heroin, an opiate. Another difference concerning the number of days spent on illegal activities for profit is probably related to the higher price of heroin. The current price of heroin on the illicit market is between 50 and 160 Euros per gram, depending on quality, which should be compared to a common synthetic opioid such as fentanyl which has a price varying between 15 and 30 Euros per patch (personal communication with the police, June 2015).

Other findings are that a higher number of those with mainly opiate use have also used cocaine and cannabis, and they have a longer duration of cannabis and amphetamine use compared with the “other opioids group”. Only the two latter findings were confirmed after Bonferroni-Holm correction. These findings, however, refer more to the participants’ drug history than to the severity of their current problems. Of special interest then are the indications of hazardous use. The opiate group more often report that they inject the drugs, but three out of four in the other opioid group also report that they inject. Injection is an important indication of serious problems, more important than the substance involved [36, 37]. In both groups, more than nine out of ten use multiple substances in combination the same day, both groups had experienced multiple overdoses, and both groups have been treated several times in detox as well as for alcohol and drug problems. Both groups have extensively used "other opiates and painkillers” as well as “medicines/pills (sedatives)”, as labelled in ASI. The type of opioid used may partly depend on the local illicit market supply. And as mentioned in the Methods section, the opiate users were more often than opioid users migrants from areas where heroin misuse was earlier established. The tendencies however are similar to elsewhere, i.e. opioids that may be prescribed as medicines are also more frequent in irregular use [5–7].

Another explanation for the extended use of other opioids may be that those who had not been admitted to OST programs may have initiated an “OST on their own”, i.e. used illicit buprenorphine or methadone as self-medication to avoid continued adverse consequences of using heroin and other opiates/opioids. There is a difference between the groups concerning one specific health problem, since opiate addicts more often suffer from hepatitis C. This difference in percentages infected, partly relates to the difference in injection rate. But there may be more factors involved. One factor is the onset time of intravenous administration, since early onset time gives more possibilities to be infected, and not just how often they now inject. Another factor is related to geography. In areas were Hep C is less spread, the risk of being injected is less.

Looking at the total health picture, the similarities are much greater, with all groups having severe somatic as well as psychiatric health problems, with additional severe problems in social situations. Thus, the study shows that both groups in this study have extensive problems, and that similarities between the groups are much greater than differences. This is the first study comparing the severities of problems between groups of opioid users, based on the Swedish categorization of opioid used, i.e. opiates vs other opioids. Based on the results of ASI assessment, we find no reasons for the categorization in the two groups as a bases for eligibility to OST.

### Methodological discussion

There is a need to discuss whether these findings are credible, reliable and relevant. ASI interviews were used in different versions from different years and authorities, but since only items with the same or very similar wordings were used, this should not have affected the validity. The ASI uses a categorization of drugs that is not consistent with the categorization of opiates and other opioids. Only heroin, methadone and Subutex are specified in the ASI questions. Other drugs based on the same generic substances as Subutex (i.e. buprenorphine) go into the drug category of “other opiates/painkillers” along with morphine, opium and fentanyl. Those variables from the ASI interview therefore have limited validity for categorizing drugs into groups (opiates vs. other opioids) relevant for the application of the statutes (SOSFS 2009:27 (M)). Since our categorization instead is based on the more accurately rendered OST assessment, that above-mentioned problem does not affect the present study. Data from the retrospective interviews were compared with the information in the OST-investigation and the hospital records to validate the material. The only difference, which concerned number of days using in a month, can be interpreted as lack of precise memory. That specific bias, however, does not affect the findings of the study, since retrospective interviews were used with both groups. Repeating comparisons between the drug type groups without using the retrospective data did not change the findings.

### Implications

Since there are no previous studies comparing problem severity in opioid users between primary opiates vs. other opiates used, this study may have some bearing on legal policies. Similarities between the groups are striking, and the few differences found do not seem to provide a rationale for different evaluations of the need for treatment. Injection use seems to be more problematic among the opiate users, but is also highly frequent among the other group explored here. Hepatitis C is indeed a dangerous condition, but suicidal acts and using multiple substances both constitute threats to the person’s life and occur in both groups. These aspects may have an impact on the kind of supplementary treatment needed.

## Conclusions

Both groups assessed for OST– those who had used opiates and those who had used other opioids – were found to have extensive problem severity in all seven ASI areas and should be considered as a heavy drug use population with similar needs for treatment and care. Since there are no previous studies conducted in a natural setting, comparing problem severity in opioid users between primary types of opioid used, this study may have some bearing on the legal policies.
